# Supplementary material for: MetaRibo-Seq measures translation in microbiomes
Source: Nat Commun. 2020 Jun 29;11:3268. doi: 10.1038/s41467-020-17081-z (PMC7324362; doi:10.1038/s41467-020-17081-z)
Supplement: Supplementary file 10 — Supplementary Data 7 [file 41467_2020_17081_MOESM10_ESM.zip › File2/Confidence_High_Taxonomy/198037_out.krona.html]

Javascript must be enabled to view this page.

members
magnitude
magnitudeUnassigned
count
unassigned
taxon
rank

198037\_out

3

2
3
superkingdom

976
3
phylum

class
200643
3

171549
3
order

family
3
815

3
816
genus

1262742
3

SRS893292\_contig\_number\_3147SRS893295\_contig\_number\_13030SRS893300\_contig\_number\_6648
species
